# PopPlanner: visually constructing demographic models for simulation

**DOI:** 10.3389/fgene.2015.00150

**Published:** 2015-04-23

**Authors:** Gregory B. Ewing, Pauline A. Reiff, Jeffrey D. Jensen

**Affiliations:** School of Life Sciences, École Polytechnique Fédérale de LausanneLausanne, Switzerland

**Keywords:** population genetics, coalescent, simulation, demographics

## Abstract

Currently there are a number of coalescent simulation programs that support a wide range of features, such as arbitrary demographic models, migration, and sub structure. Defining the model is done typically with either text files or command line switches. Although this has proven to be a powerful method of defining models of high complexity, it is often error prone and difficult to read without familiarity both with command lines and the program in question. A intuitive GUI based population structure program that can both read and write applicable command lines would dramatically simplify the construction, modification, and error checking of such models by a wider user base.

**Results:** PopPlanner is a tool to both construct and inspect complicated demographic models visually with a GUI where the user's primary interaction is through mouse gestures. Because of their popularity, we focus on ms and by extension msms, command line coalescent simulation programs. Our program can be used to find errors with existing command lines, or to build original command lines. Furthermore, the graphical output supports a number of editing and output features including export of publication quality figures.

## Background

**Figure 1:**
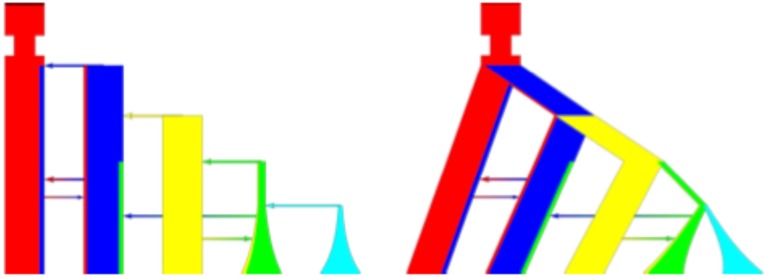
**Complicated command line export**. Example of different graphical representations. Left is the column representation while right is the tree representation. The command line is: ms 10 1 –t 5 5 5 0 0 0 –m 1 2 162.0 –m 1 2 162.0 –m 2 1 247.0 –m 3 4 213.0 –m 4 2 216.0 –g 4 1.61 –g 5 2.354 –en 0. 988 4 0.2 –ej 0.991 5 4 –ej 1.652 4 3 –ej 2.336 3 2 –ej 3.09 2 1 –em 3.233 1 0.52948 –en 3.542 1 1.0.

Simulation of populations with non-trivial demographic histories is frequently completed with coalescent simulators for performance reasons, with respect to both memory and speed. There are a number of coalescent simulators currently available (e.g., Hudson, 2002; Ewing and Hermisson, 2010; Excoffier et al., 2013, for a review see Arenas, 2012). Unfortunately, specifying complicated models is itself complicated. Currently programs use either a text file to specify the model (Excoffier et al., 2013) or a command line (Hudson, 2002; Ewing and Hermisson, 2010). The most popular appears to be ms command line format and many manuscripts will often provide the command lines used in their studies. Currently, ms command lines must be manually constructed, which is time consuming, error prone and particularly difficult for users not familiar with command line interfaces. For a typical example see Figure 1's caption.

To address these issues, we have developed a GUI tool called PopPlanner for graphically constructing complicated demographic models. It has been designed and tested to be easy to use without extensive reading of a manual and with minimal knowledge of ms command lines. However some knowledge of standard population genetic definitions and conventions is assumed. PopPlanner permits the incremental construction of complicated models in a straightforward way, where many parameters can be adjusted via dragging the relevant feature in the window, or editing a number in a text field. Other visual adjustments can be made and saved and several different export formats are available (png, eps).

## Implementation

PopPlanner is an intuitive visual editor where the majority of editing is with interactive mouse gestures. The basic editing flow still closely follows the implied representation in ms. In particular the user edits parameters by adding “events” which corresponds to flags in the command line. This allows PopPlanner to model anything that is possible in ms and ensures that in the future various extensions, such as selective sweeps in msms, can be added with minimal development effort. The basic view is shown in the left panel of Figure 1. Each deme is represented as a column with customizable colors. Migrations are shown as graded color arrows, and population splits and joins as solid color arrows. Each population is represented as a color, and the extent of migration to another population is visualized as the proportion of that color found in the migrant population representation. This view closely follows the internal representation that is used by both ms and msms. However, often a tree view is preferred, as is shown in the right panel of Figure 1. It is possible to define un-tree like models in ms but a majority of models fit the tree style well in practice.

The program is implemented in the Java programming language and allows easy deployment on any platform that supports Java (Windows, OSX, Linux, Sun OS). Internally we have used the standard Model View Controller pattern as often as practical, as is considered to be best practice for such applications. This means that each portion of code tends to only be associated with one aspect of the program so that later code modifications have minimal unintended consequences.

## Results

The user begins by specifying the initial number of populations at sampling time where time goes pastward. The initial state is the number of populations and number of samples from each population. Once a base model is defined, it can then be edited either with mouse gesture or by using the toolbar. For example, to adjust the population size event of a population, the user can click on that population in the editing window and can then enter a value or manipulate the value slider. Alternatively one can edit the time of the event by dragging up and down. Some items have purely graphical data that can be edited, making the graphics more customizable, without affecting the model. For example, migration arrows can be moved up and down as to avoid overlaps, or population join events can be moved left/right in the tree view mode to permit more pleasing tree views.

A second way of using PopPlanner is to inspect an existing command line, or by writing a command line interactively. By entering a command line, the graphical representation is updated, which can then be edited as before. The graphical representation of a model can be exported in either PNG or EPS format for high quality import into other programs.

As the program is running there are several features that enable ease of use without extensive knowledge of either ms or command lines. The command line is interactively updated and thus it is easy to see what options are changing when sliders or other items are dragged. There is an area indicating the last error, which makes a best attempt to inform the user of why some parameter change is not possible. Finally, there is a help message area where hints on what the user may want to do based on the correct selection state of the program.

### Detailed example

**Figure 2:**
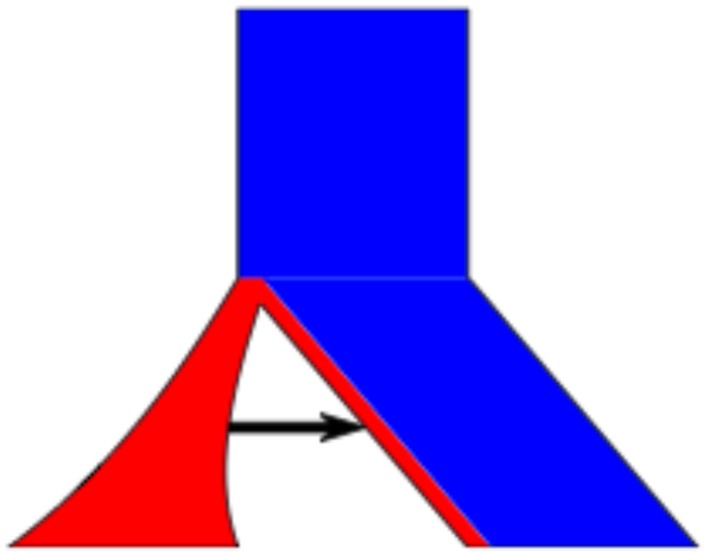
**Detailed Example**. The example population model created in the text. The command line is ms 10 1 –I 2 5 5 –m 1 2 201.5 –g 1 1.0 –ej 2.0 1 2.

To illustrate the ease of editing a population, we will give a simple example shown in Figure 2. Here we start with 2 populations, where population 1 joins population 2 at time t2, and population 1 (red) being half the initial size of population 2 (blue). Additionally, population 1 experiences exponential growth and there is migration from population 1 to 2.

1. We start with an initial population count of 2 in the initial state subwindow. This is the default starting population.
2. Click population 1 (left most) and then population 2 (right most). Note that both populations are “selected.” If they are not already joined we click on the “Join” button.
3. Click on the Join arrow that was just created: it should be solid red. The event subwindow on the right will now be editable. Change the value to 2 and press enter, or drag the arrow.
4. Click population 1 and then click on the “Growth” button. Note that the start of exponential growth is currently where the last mouse click was. Dragging in population 1 above the start of the growth will move it “down” to present time.
5. Again click population 1 then 2. Add a migration event. The user may need to change the scale of the migration arrows with the dialog under the Options menu called “arrows.”
6. Click population 2 and add population size. Set its value to 0.5.
7. For nicer results change the view to a “tree” style under the Options menu.

**Figure 3:**
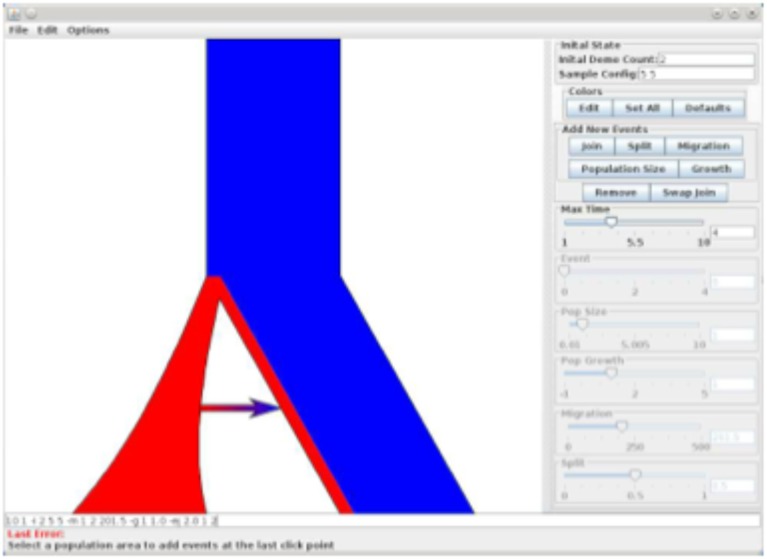
**Screen shot of PopPlanner A graphical description of the program showing relevant features**.

The results and command line are shown in Figure 2 (and a screenshot of the interface used to construct this model is shown in Figure 3).

## Conclusions

ms and msms are widely used and powerful coalescent simulators that require complicated and error prone command lines to define complex population models. PopPlanner is a program that will significantly simplify construction of these command lines by both allowing graphical construction of complicated models, or importing existing command lines for easy modification and verification. PopPlanner requires minimal expertise to use and includes a number of useful features such as figure export and interactive modification of models. These features will expand the user base of both ms and msms and make using existing models in manuscripts simpler with command line import.

## Availability and requirements

Project Name: PopPlanner
Project Home Page: http://jensenlab.epfl.ch/
Operating systems: Linux, Windows and OSX.
Programming Language: Java
Other requirements: Java 1.6 or better.
License: LGPL 2 or higher.
Any restrictions to use by non-academics: No

## Author contributions

PR coded the original prototypes and gui programming. GE wrote the final code. GE and JJ wrote and edited the manuscript.

### Conflict of interest statement

The authors declare that the research was conducted in the absence of any commercial or financial relationships that could be construed as a potential conflict of interest.
